# Mesozooplankton Grazing on Picocyanobacteria in the Baltic Sea as Inferred from Molecular Diet Analysis

**DOI:** 10.1371/journal.pone.0079230

**Published:** 2013-11-18

**Authors:** Nisha H. Motwani, Elena Gorokhova

**Affiliations:** 1 Department of Systems Ecology, Stockholm University, Stockholm, Sweden; 2 Department of Applied Environmental Sciences, Stockholm University, Stockholm, Sweden; College of Charleston, United States of America

## Abstract

Our current knowledge on the microbial component of zooplankton diet is limited, and it is generally assumed that bacteria-sized prey is not directly consumed by most mesozooplankton grazers in the marine food webs. We questioned this assumption and conducted field and laboratory studies to examine picocyanobacteria contribution to the diets of Baltic Sea zooplankton, including copepods. First, qPCR targeting ITS-1 rDNA sequence of the picocyanobacteria *Synechococcus* spp. was used to examine picocyanobacterial DNA occurrence in the guts of Baltic zooplankton (copepods, cladocerans and rotifers). All field-collected zooplankton were found to consume picocyanobacteria in substantial quantities. In terms of *Synechococcus* quantity, the individual gut content was highest in cladocerans, whereas biomass-specific gut content was highest in rotifers and copepod nauplii. Moreover, the gut content in copepods was positively related to the picocyanobacteria abundance and negatively to the total phytoplankton abundance in the water column at the time of sampling. This indicates that increased availability of picocyanobacteria resulted in the increased intake of this prey and that copepods may rely more on picoplankton when food in the preferred size range declines. Second, a feeding experiments with a laboratory reared copepod *Acartia tonsa* fed a mixture of the picocyanobacterium *Synechococcus bacillaris* and microalga *Rhodomonas salina* confirmed that copepods ingested *Synechococcus*, even when the alternative food was plentiful. Finally, palatability of the picocyanobacteria for *A. tonsa* was demonstrated using uptake of ^13^C by the copepods as a proxy for carbon uptake in feeding experiment with ^13^C-labeled *S. bacillaris*. These findings suggest that, if abundant, picoplankton may become an important component of mesozooplankton diet, which needs to be accounted for in food web models and productivity assessments.

## Introduction

The smallest photosynthetic organisms include autotrophic picoplankton, a diverse group united by size <2 µm. This group contributes as much as 40% of global ocean primary productivity and is mainly composed by picocyanobacteria [1], [2]. In marine environments, picocyanobacteria encompassing diverse strains are represented by the genera *Synechococcus* and *Prochlorococcus* [1]–[3], with the former being the major contributor to the total photosynthetic biomass in the temperate oceans [1], [4]. Similar to other marine areas, *Synechococcus*-type strains dominate Baltic Sea picocyanobacteria [5] that contribute up to 50% of total phytoplankton biomass [6] and up to ∼70% of total chl *a* during summer [7], [8] in offshore Baltic Sea waters.

Although much of biomass and primary production, particularly in low productive systems is due to the picoplankton, this phytoplankton fraction is considered largely unavailable for most metazooplankton, with heterotrophic nanoflagellates and ciliates being the major grazers on pico-sized prey [2], [9]. Among metazooplankton, appendicularians [10], cladocerans [11], [12], rotifers [12], and bivalve larvae [13] are known to substantially feed on picoplankton, but not copepods. These most important grazers in marine systems do not feed efficiently on particles of this size as shown by feeding experiments with algal cultures [14] and natural phytoplankton assemblages [15]. The size of the smallest algae that a filtrator can capture is a function of the distance between the setules on the filtering appendages, whereas the maximum size of ingestible particles is generally determined by the grazer body size [16]. Colony-building picoplankton can easily be grazed by crustacean zooplankton [2], [9], while single-celled species <2 µm would be too small to be efficiently retained by most of mesozooplankton filtrators [14]–[17]. Therefore, mesozooplankton grazing on picoplankton is generally considered to be non-efficient or intermittent [16], [17]. Nevertheless, it has been suggested that “picocyanobacteria are in a size range suitable for utilization by nauplii and early copepodite stages as well as rotifers” [2], and some field and experimental studies indicate that ingestion of unicellular picoautotrophs by copepod species does occur [17], [18]. In many systems, under food limiting conditions, feeding on picoplankton would be an advantage for grazers. It has been hypothesized that at low concentrations of phytoplankton, zooplankton grazers reduce their energy expenditure or even stop feeding [19]. Also, at low concentrations of preferred phytoplankton species, zooplankton may switch to more abundant suboptimal prey [20].

Most of what we know about prey size selectivity in zooplankton is derived from feeding experiments that involve bottle incubations and analysis of the prey disappearing from the media, gut fluorescence measurements of grazers, and radioactive labeling [16]. Of these approaches, only gut fluorescence method is applicable for field studies and has been used to detect and quantify picocyanobacteria in copepod gut contents [18]. Grazing on picoplankton has also been studied using other techniques, such as fluorescent labeled cells, metabolic inhibitors, dilution technique, flow cytometry and radioisotope-labeled prey [9]. General pitfalls associated with these methodologies are the “bottle effects” and improper controls which do not correct for nutrient regeneration by zooplankton, resulting in underestimation of grazing rate and misinterpretation of selective feeding [21], [22]. Moreover, many of the early isotope studies were not accurate because of recycling of the isotopes as a result of the excretion and respiration by phytoplankton and zooplankton [23]. There are also sources of error involved with gut fluorescence measurements and calculations of both ingestion and filtration rates that, particularly with fast growing picoplankton, can lead to underestimation of the grazing impact due to breakdown of pigment during digestive activity [24].

Currently, studies on trophic relationships are rapidly turning to DNA-based techniques [25]. Molecular methods based on quantitative PCR (qPCR) that can both identify prey of interest and quantify its contribution to the stomach content have been recently applied for diet analysis in zooplankton, including copepods [26]–[28]. The approach is particularly relevant for detecting prey groups with variable morphological characters and pigment composition, such as picocyanobacteria [29]. In qPCR-based diet analysis, there are a few molecules that are particularly informative for target identification and quantification (e.g., 16S rDNA, cytochrome *c*, and nuclear ribosomal genes and their spacers) [25]. The high abundance of these genes makes them an attractive target in molecular diet analysis [26]–[28], [30], but also adds difficulty to account for copy number variability per cell in response to environmental conditions and strain composition in wild populations [31]. Another technical difficulty inherent to molecular diet studies on microscopic aquatic animals is to control for non-ingestion contamination by the target prey [27] that can be present in carry over water and/or adhere to body surfaces of the animal.

In line with the current views on mesozooplankton ability to graze on picoplankton, we hypothesized that in the Baltic plankton communities, picocyanobacteria are consumed mostly by nauplii, rotifers and cladocerans, but not by larger copepodites [2]. To test this hypothesis, we used molecular diet analysis based on qPCR targeting ITS-1 sequence of the picocyanobacteria *Synechococcus*. Using this technique, we quantified picocyanobacterial DNA in the guts of different zooplankters (rotifers, cladocerans and dominant copepod species at various developmental stages) collected during the growth season in a coastal area of the northern Baltic proper. Furthermore, the amount of picocyanobacteria in the guts was related to the ambient *Synechococcus* spp. and phytoplankton abundances. We also conducted feeding experiments with laboratory reared copepod *Acartia tonsa* fed picocyanobacterium *Synechococcus bacillaris* and cryptophyte *Rhodomonas salina* to (1) test whether copepods ingest picocyanobacteria in the absence of protozoan grazers; (2) determine the non-consumptive contribution of picocyanobacteria to zooplankton samples, due to adherence to body surfaces and other sources of contamination; and (3) quantify carbon uptake from the picocyanobacteria in the copepods using ^13^C-labeled *S. bacillaris* as prey.

## Materials and Methods

### Ethics Statement

The sampling was conducted within national Swedish monitoring in the coastal waters of Sweden and no specific permissions were required for the sampling locations of this study. Also, we did not require ethical approval to conduct this study as we did not handle or collect animals considered in any animal welfare regulations and no endangered or protected species were involved in the samplings or the experiments.

### Field zooplankton collections

Zooplankton samples were collected in the Himmerfjärden Bay, a coastal area of the northern Baltic proper (59°00′ N; 17°43′ E, bottom depth ∼28 m). Samples were collected around noon, bi-weekly, July to September 2008, by vertical bottom to surface tows using a 90 μm WP-2 net (diameter 57 cm). From each tow, randomly selected zooplankton were preserved in bulk using RNA*later* and stored at −20°C for ∼2 years [32]. From these samples, different species and developmental stages of mesozooplankton were picked under a dissecting microscope with forceps, rinsed in artificial seawater, and transferred in groups (7–10 ind sample^−1^ for crustacean zooplankton and 12–25 ind sample^−1^ for rotifers) into 1.5 ml Eppendorf tubes for DNA extraction. The following species/groups were selected for the analysis: (1) copepodites (CII–VI) of *Acartia* spp. and *Eurytemora affinis*, (2) cladoceran *Bosmina maritima*, (3) podonids (mixed samples for *Podon intermedius* and *P. leuckartii*), (4) copepod nauplii (stages N1–N6; mixed samples for *Acartia* spp. and *E. affinis*), and (5) rotifers (mixed samples for *Synchaeta* spp., *Keratella quadrata*, and *K. cochlearis*). To prepare reference samples (contamination control), freshly hatched *Artemia* spp. nauplii (San Francisco Bay Brand; 10 ind sample^−1^) were used and treated in the same way as the zooplankton samples.

### *Synechococcus* in the water column

Phytoplankton were collected with a plastic hose (inner diameter 19 mm) as integrated water samples (0–14 m) on the same occasions as zooplankton. The samples were immediately pre-filtered with a 35 µm sieve to remove large plankton and 100–250 ml of the filtrate were concentrated onto a 0.2 µm nylon membrane (47 mm diameter; Millipore™). The filters were folded, transferred in the 1.5 ml Eppendorf tubes, and stored at −80°C until further analysis.

### DNA extraction

Zooplankton samples were incubated in 40 µl of 10% Instagene Chelex (Bio-Rad) for 30 min at 105°C [33]. After centrifugation (12 000×*g*, 5 min), the supernatant (30 μl) was transferred to a clean Eppendorf tube and stored at 4°C for 1–2 days. To extract DNA from the filters with phytoplankton assemblages, four sections from each filter were excised with 7 mm diameter punch and disrupted using Fast Prep® instrument and glass beads (<106 µm, Sigma-Aldrich) for 40 s. DNA was subsequently extracted with 400 µl of 10% Instagene Chelex-100 as described above, intermittently mixing the tube manually. The DNA measurement and quantification of *Synechococcus* spp. by qPCR were conducted using the same protocol as for the zooplankton samples. Concentrations of DNA (7.5–110 ng µl^−1^) and purity were determined with a Nanophotometer^™^ (Implen); *A*_260_/*A*_280_ varied from 1.8 to 1.9.

### Quantification of *Synechococcus* in zooplankton guts and water samples

To quantify *Synechococcus* spp. in zooplankton samples and plankton assemblages collected on the filters, a qPCR assay was applied using universal primers specific for *Synechococcus* (P100A: 5′ ggt tta gct cag ttg gta gag cgc 3′; P3: 5′ ttg gat gga ggt tag cgg act 3′) and hydrolysis probe (S100A: 5′ FAM- ctt tgc aag cag gat gtc agc ggt t- TAMRA 3′) targeting the ITS-1 sequence spanning between 16S rDNA and 23S rDNA genes in the ribosomal operon of *Synechococcus* spp. [34]. These primers and probe have been broadly tested for their ability to amplify different *Synechococcus* strains from five different lineages using a 16S rRNA inferred phylogenetic tree; the strains were isolated from various fresh and brackish waters, including the Baltic Sea [34]–[36]. A synthetic DNA oligonucleotide (Invitrogen Ltd.) comprising 75 bp of the target sequence (*Synechococcus* sp. BS20: positions 1868–1942; 5′ ggt tta gct cag ttg gta gag cgc ctg ctt tgc aag cag gat gtc agc ggt tcg agt ccg cta acc tcc atc caa 3′) was used as a standard [37]. Standard curves were generated using a five step 10-fold dilution series, 1.1×10^3^–1.1×10^7^ target amplicons per reaction; triplicate no template controls (NTC) were included in all runs. Reactions were performed in triplicate using the TaqMan Gene Expression Master Mix (Applied Biosystems) and a StepOne real-time cycler (Applied Biosystems). Amplifications were performed in a 20 μl reaction mixture as follows: 2 minutes at 50°C, 10 minutes at 95°C, and 40 cycles of 15 seconds at 95°C and 1 minute at 60°C. Threshold cycle (C_t_) was set automatically by StepOne Software 2.0. For each standard curve, the *r^2^* value, the amplification efficiency (*E%*) and the *y*-intercept value were recorded. Coefficient of variation was used to estimate intra- and inter-assay variability [38]; see Table S1 for statistical evaluation of efficiency and repeatability of the qPCR assays. PCR products were also visualized in the GelDoc after electrophoresis at 90 V for 1 h on 2.5% agarose gel prepared in 1× TAE buffer containing 0.5 mg ml^−1^ ethidium bromide.

In the test samples, *Synechococcus* spp. ITS-1 copy number was estimated from the standard curves. The molecular weight of the standard was used to calculate ITS-1 gene copy number per reaction:
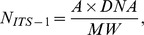
where; *N_ITS-1_* is number of copies (µl^−1^), *A* is 6×10^23^ is the gene copies mol ^−1^ (the Avogadro constant), *DNA* is DNA concentration (g µl^−1^), and *MW*– molecular weight of the amplicon, 46228 g mol^−1^ [39].

### Total phytoplankton

Sampling and analysis of phytoplankton were conducted as a part of Swedish National Monitoring Programme, following HELCOM guidelines [40]. Briefly, samples were settled in Utermöhl chamber and examined using a NIKON inverted microscope with phase contrast. Phytoplankton (>2 μm; ≥500 cells) were counted in diagonals or on the half/whole chamber bottom at 150× and 400× magnification;. cell volume was calculated from size measurements (≥25 cells species^−1^).

### Non-ingestion contamination by picocyanobacteria (Experiments I and II)

To determine the amount of *Synechococcus* that might have been attached to external body parts of copepods, but not ingested, feeding experiments were conducted with the copepod *Acartia tonsa* reared in the laboratory and axenic cultures of *Synechococcus bacillaris* (CCAP 1479; cell size: 2 µm) and *Rhodomonas salina* (strain CCAP 978/24; cell size: 8 µm) as food; the latter alga is a high quality food commonly used in experiments with *Acartia* [27]. The picocyanobacterial and algal concentrations (cells ml^−1^) were determined using a haemocytometer and converted to carbon mass [41]. As *Synechococcus* has been reported to build colonies and aggregates [42], the cultures were pre-filtered using 20 µm sieve and the number of cells associated in microcolonies was noted (mean ± SD: 1.8±0.3%; n = 5) when determining cell concentrations. To relate cell number to copy number of ITS-1 in the standard, DNA was extracted from 200 µl of *S. bacillaris* culture with known cell density using Chelex method and analyzed by qPCR in the same way as the copepod samples.

Older copepodites (CIV–V; thereafter referred to as adults) and nauplii were used as test animals. The adults were picked using a wide mouth pipette and incubated in artificial seawater (7 PSU, 18°C) for 8 h without food. To obtain nauplii, eggs were collected from the batch cultures and incubated in 96-well microplate in the sterile artificial seawater. Starved adults (experiment I) and nauplii (experiment II) were randomly assigned to two treatments: (1) dead controls (22–30 and 10 ind sample^−1^ for adults and nauplii, respectively); newly hatched nauplii (non-feeding stage) were used in this treatment to ensure empty guts; and (2) fed adults (13–15 ind. sample^−1^) and nauplii at the first feeding stage NIII (10 ind. sample^−1^). To prepare dead controls, all animals were killed by immersing in 95% ethanol prior to exposure to *Synechococcus* to prevent ingestion. In all experiments and treatments, copepod groups were placed in 50 ml chambers with false bottoms (mesh size 60 and 20 µm for adults and nauplii, respectively) to prevent ingestion of fecal pellets by live copepods, and exposed to a mixture (1∶6 by carbon content; 0.25 mg C l^−1^) of *Synechococcus* (1.5×10^5^ cells ml^−1^) and *Rhodomonas* sp. (7.4×10^5^ cells ml^−1^); these prey densities were selected to approximate summer phytoplankton community in terms of the proportion between the picoplankton and larger phytoplankton fractions in the Baltic Sea [6]. The exposure lasted 3 h; this time was considered sufficient for the copepods to recover from handling, start feeding normally and fully fill their guts. Upon termination of the experiment, the copepods were collected on the 20 µm sieve, washed twice in 7 PSU artificial sea water and transferred with forceps to Eppendorf tubes with RNA*later*. The samples were then processed in the same way as the field samples.

### Carbon uptake by copepods fed picocyanobacteria (Experiment III)

The uptake of carbon from picocyanobacteria by copepods was measured using *S. bacillaris* labeled with ^13^C and fed to *A. tonsa* copepodites. The ^13^C-labeled *Synechococcus* was prepared by replacing NaH^12^CO_3_ with NaH^13^CO_3_ in the f/2 medium that was done by adding 2 ml of a NaH^13^CO_3_ stock solution (336 mg NaH^13^CO_3_ in 100 ml H_2_O sodium bicarbonate, ^13^C, 99%, Cambridge Isotope Laboratories) per 100 ml of the medium and growing the culture for 4 days in a climate room at 18–20°C at constant illumination. The labeling resulted in isotope signatures (δ^13^C) of −13.6‰ and 6683.4‰ for untreated and ^13^C enriched cultures, respectively. To measure the ^13^C uptake by the copepods, 25–30 copepodites (CIII–CIV) of *A. tonsa* were assigned to two treatments, each in three replicates: (1) dead controls that were prepared as described in experiments I and II and incubated with the ^13^C-labeled picocyanobacteria for 4 h, and (2) fed copepods that were incubated for 96 h. In addition, animals sampled at time 0 were used to measure the carbon signature before feeding on the enriched material. At the end of the incubation, the copepods were rinsed with excess of Milli-Q water, and live copepods were incubated with unlabelled picocyanobacteria for another 4 h to ensure replacing of the ^13^C-labeled food in the guts. All copepods were transferred into pre-weighed tin capsules (25 ind. sample^−1^) and dried at 60°C for 24 h. The δ^13^C values in the copepods were used as a proxy for carbon uptake; these values were measured with a continuous flow isotope ratio mass spectrometer (Europa Integra) at the UC Davis Stable Isotope Facility (University of California, USA).

### Statistical analysis

Gut content (GC) in terms of *Synechococcus* ITS-1 copies ind. ^−1^ and size specific GC (ssGC; copies µgWW^−1^, where WW is zooplankter wet weight [43]) in the field-collected animals were compared among zooplankton groups and species by unpaired *t*-test with Welch's correction for unequal variances (GraphPad Prism 5.0®, GraphPad Software). The δ^13^C values of the copepods in the experiment III were compared among the treatments (i.e., start animals, dead controls and fed copepods) using one-way ANOVA followed by *a posteriori* comparisons with the Tukey HSD test. To evaluate effects of *Synechococcus* and total phytoplankton abundance on GC in the copepods, generalized linear model (GLM) with normal distribution and log-link (Statistica v. 10, StatSoft Inc.) and pooled data for *Acartia* spp. and *E. affinis* were used. The regression analysis was limited to the copepods, because the GC data for this group were available on most sampling occasions. Data were Box-Cox transformed and the residuals were linear, homogenous, normally distributed and not correlated.

## Results

### Presence and abundance of *Synechococcus* DNA in mesozooplankton

**Figure 1:**
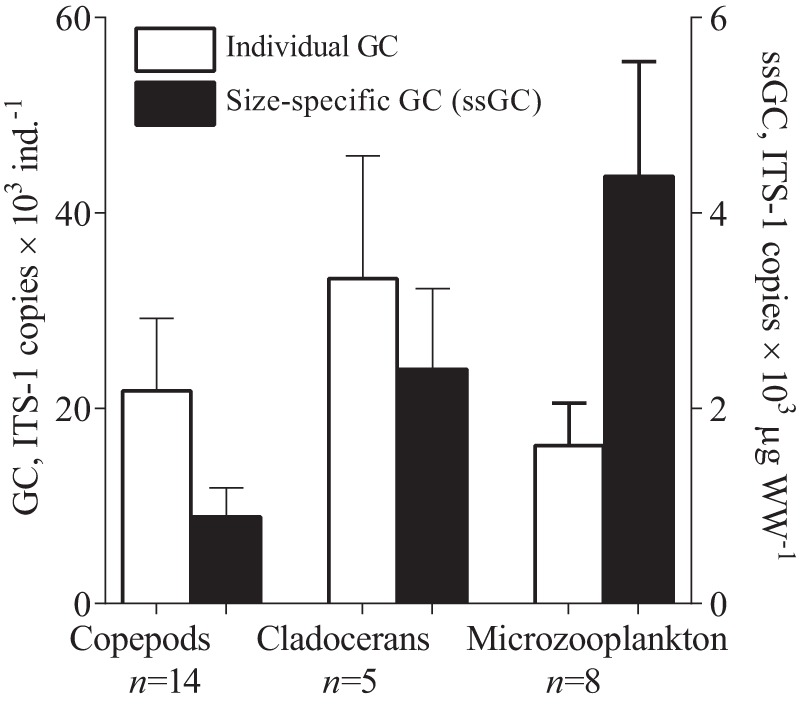
Occurrence of *Synechococcus* spp. in field-collected zooplankton. Individual gut content (GC; prey ITS-1 copies ×10^3^ ind^−1^) and size-specific gut content (ssGC; prey ITS-1 copies ×10^3^ µgWW^−1^) in main zooplankton groups: copepods (adults and older copepodites of *Acartia* spp. and *Eurytemora affinis*), cladocerans (*Bosmina maritima* and *Podon* spp.) and microzooplankton (rotifers *Synchaeta* spp., *Keratella quadrata*, and *K. cochlearis*, and copepod nauplii). Data are shown as mean ± SD, number of samples is given below the group name.

All field-collected zooplankton samples tested positively for *Synechococcus* DNA (Table 1), whereas no amplification was observed in the reference samples (newly hatched *Artemia*). The amount of ITS-1 copies varied about 7-fold (8×10^3^ to 53.8×10^3^ per zooplankter), with substantial differences between the species and groups (Table 1, Figure 1). The differences in GC between main zooplankton groups were significant: copepods vs. microzooplankton (unpaired *t*-test; t_17_ = 2.150, *p*<0.04), cladocerans vs. microzooplankton (t_7_ = 3.891, *p*<0.006), and copepods vs. cladocerans (t_6_ = 2.403, *p*<0.05). For ssGC, a different pattern was observed, with the values decreasing with the body size and differing significantly between the zooplankton groups: copepods vs. microzooplankton (t_17_ = 12.507, *p*<0.0001), copepods vs. cladocerans (t_6_ = 5.60, *p*<0.0014), and cladocerans vs. microzooplankton (t_7_ = 3.402, *p*<0.0145; Figure 1).

**Table 1:** *Synechococcus* abundance (ITS-1 copies ×10^3^ ind^−1^) detected in different mesozooplankton species/groups.

Species/group	Category	*Synechococcus* mean (min–max)	*n*
*E. affinis,* adults	copepods	21 (16–31)	7
*Acartia* spp., adults	copepods	23 (11–36)	7
*Acartia* spp., *E. affinis*, copepodites (CII–VI)	copepods	21 (17–24)	2
*Acartia* spp., *E. affinis,* nauplii (N1–N6)	microzooplankton	18 (15–24)	5
*B. maritima* (0.4–0.6 mm)	cladocerans	23 (20–27)	2
podonids	cladocerans	35 (24–54)	4
rotifers	microzooplankton	10 (8–12)	3
Nauplii and rotifers	microzooplankton	8	1

Field-collected samples were used for the analysis. *E. affinis* – *Eurytemora affinis*, *Acartia* spp. – *Acartia bifilosa* and *A. longiremis*, *B. maritima* – *Bosmina maritima*, podonids – *P. intermedius* and *P. leuckartii*, rotifers – *Synchaeta* spp., *Keratella cochlearis* and K. *quadrata*; nauplii – *Acartia* spp. and *E. affinis*; *n* – number of samples analyzed.

### Changes in total phytoplankton and picocyanobacteria during the season

During July– September 2008, the ambient *Synechococcus* spp. abundance in terms of the number of ITS-1 copies varied from 2.2×10^5^–7.6×10^5^ copies ml^−1^, with the highest values observed in August (monthly average 4.9×10^5^ copies ml^−1^). Total phytoplankton biovolume ranged from 0.4 to 1.5 mm^3^ ml^−1^, with the peak observed at the end of the August (1.5 mm^3^ ml^−1^).

### Relationship between picocyanobacteria intake and their abundance

There were no significant differences in the GC between the copepodites of *E. affinis* and *Acartia* spp. on each sampling occasion (t_4_ = 1.70, *p*>0.05). Hence, these species were pooled for GLM relating individual GC to *Synechococcus* abundance (ITS-1 copies ml^−1^) and total phytoplankton (biovolume, mm^3^ ml^−1^). In this model, the amount of *Synechococcus* DNA in the copepod gut was positively related to the picocyanobacteria abundance and negatively to the total phytoplankton stocks at the time of sampling (Table 2).

**Table 2:** Statistical summary of the generalized linear model examining effects of *Synechococcus* abundance (ITS-1 copies ×10^3^ ml) ^−1^ and total phytoplankton (>2 µm) biovolume (mm^3^ ml^−1^) in the water column (0–14 m) on the abundance of *Synechococcus* DNA in copepod stomachs (ITS-1 copies ×10^3^ ind^−1^).

	Estimate	Standard error	Wald statistic	*p*-value
Intercept	0.243	0.332	0.535	0.464
*Synechococcus*	0.238	0.065	13.53	**0.000**
Total phytoplankton	−0.474	0.136	12.03	**0.001**

Data are Box-Cox transformed, significant effects are in bold face.

### Experiments I and II

**Figure 2:**
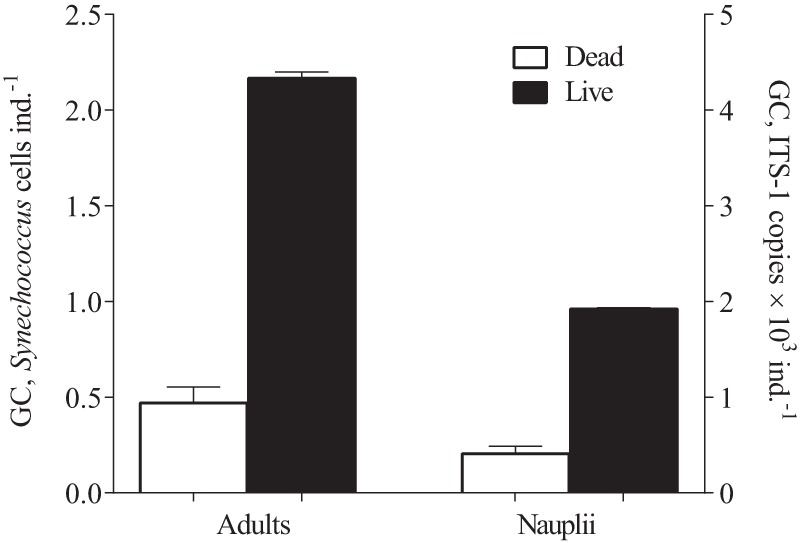
Quantities of *Synechococcus bacillaris* (ITS-1 copies ×10^3^ ind^−1^and cells ×10^3^ ind^−1^) detected in the live and dead individuals of the copepod *Acartia tonsa* (adults and nauplii) exposed to the picocyanobacterium in the feeding experiments (Experiments I and II). Data are shown as mean ± SD, *n* = 3 in all cases.

*Synechococcus* DNA were detected in both killed and live copepods exposed to the experimental feeding media (Figure 2), with values being ∼5 fold higher in the live copepods (adults: t_4_ = 32.61, *p*<0.0009; nauplii: t_4_ = 32.73, *p*<0.0001). The percentage of *Synechococcus* measured in the dead individuals compared to the live animals of the same developmental stage was similar between the adults and nauplii, 21.8% and 21.2%, respectively. The copy number of ITS-1 per cell in the *Synechococcus* culture was 2.04±0.03 as estimated by qPCR analysis of samples with known cell abundance (7.2×10^7^ cells ml^−1^). Thus, non-ingestion background corresponded to about 2200 and 965 *Synechococcus* cells ind^−1^ for adults and nauplii of *Acartia tonsa*, respectively.

### Experiment III

**Figure 3:**
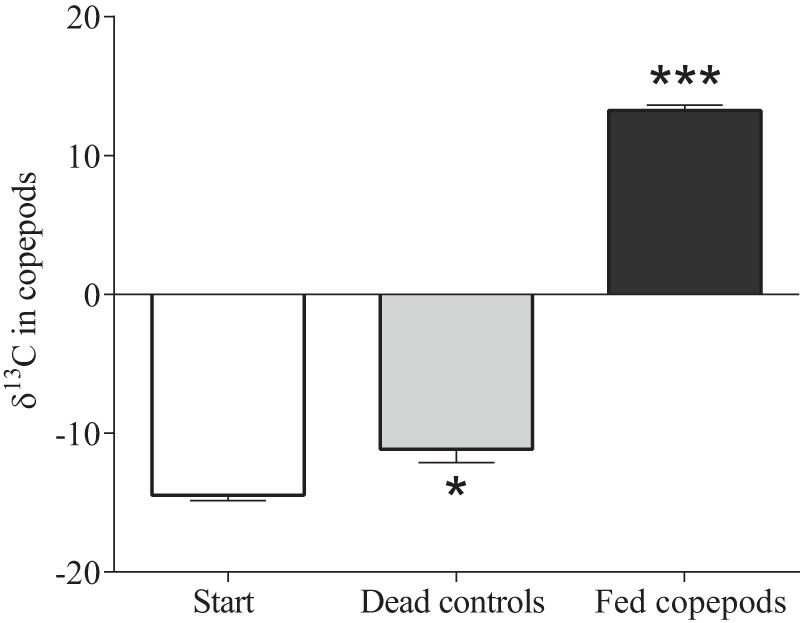
Carbon uptake from ^13^C-labeled *Synechococcus bacillaris* by the copepod *Acartia tonsa* (live and dead individuals) exposed to the picocyanobacterium (Experiment III). Carbon uptake is expressed as change in δ^13^C of the copepods from the start values. Differences between the start and each treatment group are shown by asterisks (*: p<0.05; ***: p<0.0001). Data are shown as mean ± SD, *n* = 3 in all cases.

There was a significant carbon uptake by *A. tonsa* copepodites fed *Synechococcus* (ANOVA; F = 556, *p*<0.0001; Figure 3). Moreover, measurable increase was also found in dead controls, albeit this increase was ∼10-fold lower than in the fed copepods (Tukey HSD, q = 4.305, *p*<0.05).

## Discussion

Contrary to the generally accepted view that mesozooplankton are inefficient at capturing pico-sized particles, such as autotrophic picoplankton [9], all tested species of Baltic zooplankton, including copepods of all stages, were found to directly consume substantial quantities of picocyanobacteria. Two crucial results support this conclusion. First, qPCR-based diet analysis revealed presence of *Synechococcus*, the dominant picocyanobacteria in the Baltic Sea [5], in the guts of all major zooplankton groups, including rotifers, cladocerans and copepods. Second, the feeding experiments confirmed that both nauplii and adults of *Acartia tonsa* were ingesting and assimilating *Synechococcus bacillaris* even when alternative food was plentiful and no protozoan grazers were present. The latter implies that the picocyanobacteria occurrence in the field-collected zooplankton are primarily the result of the direct grazing on picocyanobacteria and not the secondary consumption, i.e., consumption of prey that had been feeding on the picocyanobacteria. Also, the amount of *Synechococcus* adhering to the outside of zooplankters and/or caused by possible contamination during the sorting procedure was ∼20% of the total as indicated by the comparison of the picocyanobacteria abundance in the live and dead copepods exposed to *Synechococcus* in the feeding experiments. Although this indicates that gut content was the main source of the PCR-based estimates of picocyanobacteria abundance in the zooplankton samples, the non-ingestion background should be taken into consideration when analyzing zooplankton samples. The percentage of contamination was not affected by the size of the animals (adult and nauplial copepod stages), which allows applying the 0.2 correction factor for background contamination in the field samples. However, this value may depend on the ambient picocyanobacteria abundance in the water, which should be further investigated in similarly designed experiments with varying picocyanobacteria densities in the media.

As hypothesized, cladocerans, rotifers and nauplii were found to have the highest biomass-specific amounts of picocyanobacteria in their guts. These zooplankters have been reported to feed efficiently on bacteria-sized particles, including picocyanobacteria (cladocerans: [44], [45], rotifers [45] and nauplii [46]). However, contrary to our expectations and various feeding studies showing that older copepodites do not feed on picoplankton [14], [15], guts of *Acartia* spp. and *Eurytemora affinis* copepodites exposed to picocyanobacteria contained ∼2×10^4^ *Synechococcus* ITS-1 copies ind^−1^ (Table 1). According to the filtration theory, single-celled organisms <2 µm are outside of the size range of particles that copepods can retain on their feeding appendages [16], [47]. In *Acartia tonsa*, for example, the retention efficiency drops dramatically below ∼5 μm particles [48], [49]. What are then the mechanisms by which large copepods ingest relatively large quantities of *Synechococcus*? First, as mentioned above, although *Synechococcus* cells are basically solitary, they can build microcolonies with 2–50 cells colony^−1^ [42] and/or occur in loose agglomerates, particularly in summer [50]. The presence of these colonies and agglomerates in the picocyanobacteria populations would greatly increase retention efficiency for *Synechococcus*. Second, autotrophic picoplankton occurs in aggregates with detrital particles and heterotrophic bacteria, which enhances their availability for mesozooplankton [18]. Finally, copepods feeding on small particles [51] can adjust the posture of their filtering appendages and beat their maxillae at a faster rate than the same animals feeding on large particles to obtain the same ration [47].

In our feeding experiments, adult *A. tonsa* exposed to *Synechococcus bacillaris* with a very few (<2% of the total number of cells) microcolonies, consumed the picocyanobacterium, albeit at lower quantities than *Acartia* spp. (*A. bifilosa* and *A. longiremis*) in the field (∼5×10^3^ and 21×10^3^ *Synechococcus* ITS-1 copies ind^−1^ in the laboratory fed and field collected copepods, respectively). The higher abundance of picocyanobacteria cells in the guts of field-collected copepods could be related to (1) larger body size of *A. tonsa* compared to the other two *Acartia* species [43] and lower capacity for picoplankton ingestion, (2) greater picocyanobacteria aggregate formation in the field increasing retention rate, and (3) secondary consumption in the field, where feeding on protozooplankton feeding on picoplankton was likely to occur. The latter mechanism requires additional experimental studies to establish detection efficiency for DNA in the mesozooplankton guts for *Synechococcus* that underwent secondary consumption. These experimental data and the difference indicate that both solitary and grouped picoplankton cells can be ingested by the copepods and that all mechanisms outlined above may contribute to the observed variation in consumption of *Synechococcus* in the field-collected zooplankton.

Cell counts and qPCR analysis of *Synechococcus bacillaris* culture used in the feeding experiment revealed that *S. bacillaris* has two copies of ITS-1 gene per cell, which concurs with earlier observed two ribosomal operons per cell in four strains of *Synechococcus* from the Baltic Sea [36]. Assuming that in the study area picocyanobacteria populations consisted of several *Synechococcus* strains [52] and their average ITS-1 cell copy number equaled 2, we attempted to calculate the pigment-based contribution of *Synechococcus* to the gut content of the zooplankton. Using the GC data for *Synechococcus* spp. in terms of cells ind. ^−1^ and conversion factors reported in the literature: cell carbon content of picocyanobacteria of 0.25 pg cell^−1^ [41] and C:Chl *a* ratio for picocyanobacteria of 32∶1 [53], we arrived at the pigment-based equivalent of *Synechococcus* in the zooplankton guts being in the range of 0.03–0.15 ng Chl *a* ind^−1^. These values are comparable to the measured gut fluorescence 0.06–0.77 and 0.15–0.4 ng Chl *a* ind^−1^, reported for estuarine *E*. *affinis* [54] and *A*. *bifilosa* [54], [55], respectively. Thus, the contribution of picocyanobacteria in the diet of these copepods may account for 8–35% and 10–47% of copepod total gut content, respectively. Whereas the highest values are probably an overestimation related to less than full guts in the copepods, the lower end of the range, i.e., 8–10% of the total gut content would represent a conservative estimate. These values are very close to the contribution of picoplankton (∼10%) to the total carbon-based ingestion rate that has been observed in the copepods *Acartia clausi* and *Temora stylifera* in the feeding experiments using size-fractionated plankton assemblages [56]. Also, prokaryotes were found to contribute >50% to the gut content of the Baltic copepod *Limnocalanus macrurus* [57], which emphasized the possible importance of bacteria-sized particles to zooplankton diets. The comparison of *Synechococcus*-based GC with the gut pigment content measured in the Baltic cladocerans and rotifers, 0.05–1.10 and 0.05–0.37 ng Chl *a* ind^−1^, respectively [54], implies possible contribution of the picocyanobacteria being as high as 15–33%.

In our study, no significant difference in grazing on picocyanobacteria was observed between the copepods *Acartia* spp. and *E*. *affinis*, although the feeding behavior of these two species has been reported to be substantially different. For example, *Acartia* spp. often feed on specific food rather than most available food [58]. By contrast, *E*. *affinis* have opportunistic feeding mechanism; consuming smaller prey to compensate for food limitation when its preferred food is less abundant [59]. The most probable explanation for the lack of the observed differences in *Synechococcus* amounts in the guts between these copepod species is that feeding strategies in copepods may vary with food abundance in the environment [58] and that both copepods can use picoplankton as an alternative food to compensate for low phytoplankton availability. Indeed, the observed variations in *Synechococcus* GC of copepods were negatively related to availability of phytoplankton (>2 μm) and positively to the picocyanobacteria abundance (Table 2). This suggests that both copepods may increase picocyanobacteria consumption when this prey is highly abundant and when there is a food limitation. In particular, occurrence of dense blooms of filamentous cyanobacteria during summer in the Baltic Sea with concomitant decrease of edible phytoplankton [6] can substantially worsen food availability for mesozooplankton and thus contribute to the increased consumption of *Synechococcus* spp. It is also possible that zooplankton would prefer picocyanobacteria to less edible food, such as toxic filamentous cyanobacteria, particularly in the light of our findings that *Synechococcus* is digested and assimilated by the copepods. Also, the evidence is accumulating that picocyanobacteria may be a valuable nitrogen source for grazers [60]. In the Baltic Sea, the microbial food web receives substanial amounts of fixed nitrogen by diazotrophic cyanobacteria and leaking out, thus fueling microbial production including nitrogen-limited picocyanobacteria) during summer [61]. Therefore, ready access to a nitrogen source may be an important adaptive trade-off for zooplankton during periods of nitrogen limitation.

The observed feeding by mesozooplankton on picocyanobacteria has several important implications for our understanding of the marine planktonic food webs. A direct pathway of carbon transfer from picoautotrophs to metazooplankton implies a higher transfer efficiency from primary producers to primary consumers. It is commonly accepted that metazooplankton utilize bacterial carbon by preying on protozoans feeding on bacteria or ingesting detritus to which bacteria adhere [18]. However, most studies agree that picoplankton production is not efficiently transferred to metazooplankton because of the multiple trophic steps in the microbial loop [62], [63] and, consequently, energy and nutrient dynamics models [64] and budget calculations [65] have no direct bacteria → zooplankton pathway when copepods (with any demographic population structure) dominate the community. While the microbial loop pathway is by no doubt is the major energy route from picoplankton to metazooplankton in most pelagic food webs, the direct grazing by metazooplankton on picoplankton and filamentous cyanobacteria [66] may contribute measurably to zooplankton growth, particularly during periods of high picoplankton abundance and poor availability of larger phytoplankton. Although digestibility of picocyanobacteria by metazooplankton has been questioned [2], our experiments showed that *Synechococcus* was not only ingested but also assimilated by the copepods. Therefore, grazing on picoplankton by crustacean zooplankton should be more appreciated in food web models and productivity assessments. This is further supported by studies on metabolic budgets for herbivorous zooplankton showing that their daily ingestion rates on phytoplankton are insufficient to balance their respiration needs, and consumption of bacteria-sized particles may be necessary to satisfy zooplankton energy requirements [67]. Finally, ecosystem response to environmental change and cyanobacterial blooms have been suggested to increase energy flow through the microbial loop, which would decrease energy transfer efficiency to the higher trophic levels. Therefore, in the systems, where zooplankton grazers are capable to directly utilize picocyanobacterial biomass, the energy transfer from the microbial loop to the top consumers might be close to that in the classical food chain. To conclude, our findings demonstrate an important trophic link between mesozooplankton, including copepods and picocyanobacteria represented by the globally important primary producer *Synechococcus* spp. The grazing on picocyanobacteria may be a common year-round phenomenon in the Baltic Sea and, perhaps, in other aquatic environments, particularly during periods of a low food abundance. If metazooplankton grazers, particularly copepods, are capable to directly and efficiently utilize picoplankton, this would facilitate a direct energy transfer from microbial producers to metazooplankton, surpassing the microbial loop. Our results warrant a revision of current pelagic food web models linking phytoplankton to secondary production in the Baltic Sea as well as other systems where picoplankton contributes substantially to primary production.

## Supporting Information

Table S1 **Regression coefficient (** ***r^2^*** **), amplification efficiency (** ***E*** **),** ***y*** **-intercept values of the standard curves and no template controls (NTC) generated on five analytical occasions using the synthetic oligonucleotide as a standard for ITS-1 of** ***Synechococcus*** **spp.**
(DOC)
