# Supplementary material for: Mesozooplankton Grazing on Picocyanobacteria in the Baltic Sea as Inferred from Molecular Diet Analysis
Source: PLoS One. 2013 Nov 18;8(11):e79230. doi: 10.1371/journal.pone.0079230 (PMC3832457; doi:10.1371/journal.pone.0079230)
Supplement: Table S1 — Regression coefficient ( r2 ), amplification efficiency ( E ), y -intercept values of the standard curves and no template controls (NTC) generated on five analytical occasions using the synthetic oligonucleotide as a standard for ITS-1 of Synechococcus spp. (DOC) [file pone.0079230.s001.doc]

**PLoS One │ Supporting Information**

**Motwani & Gorokhova (2013)** Mesozooplankton grazing on picocyanobacteria in the Baltic Sea as inferred from molecular diet analysis

**Reproducibility and sensitivity of qPCR standard curves**

Reproducibility of the standard curves over the range of 1.1 × 10^3^–1.1 × 10^7^ amplicons of ITS-1 per reaction was high (Table S1), with no significant differences for either slopes or intercepts between the standard curves generated on five analytical occasions (slope: *F*_4,40_ = 1.17, *p* = 0.34, intercept: *F*_4,40_ = 2.37, *p* = 0.07). The amplification efficiencies and the regression coefficients were also consistently high (*E*: 96 – 101%, *r^2^* > 0.99 in all cases; Table S1). The NTC had a C_t_ value of 36.20 ± 0.68 and, therefore, the detection limit was set at C_t_ = 32.9. The intra assay variation determined using C_t_ values for duplicate samples was consistently low (CV%: 0.09–0.14%). The inter-assay CV% determined for Assays 1 and 2 using five paired samples was 0.78 ± 0.28%.

**Table S1.** Regression coefficient (*r^2^*), amplification efficiency (*E*) and no template controls (NTC) generated on five analytical occasions using the synthetic oligonucleotide as a standard for ITS-1 of *Synechococcus* spp. Intra assay coefficient of variation (CV%) for C_t_ value of field samples measured are shown as mean ± SD (*n* = 5).

|  | **Assay 1** | **Assay 2** | **Assay 3** | **Assay 4** | **Assay 5** |
| --- | --- | --- | --- | --- | --- |
| *r^2^* | 0.999 | 0.999 | 0.994 | 0.996 | 0.999 |
| *E* (%) | 99.66 | 96.84 | 100.92 | 95.68 | 98.44 |
| NTC | 36.44 ± 0.67 | 35.88 ± 0.03 | 35.18 ± 0.28 | 36.91 ± 0.03 | 36.58 ± 0.15 |
| CV (%) | 0.09 ± 0.05 | 0.09 ± 0.04 | 0.09 ± 0.06 | 0.10 ± 0.05 | 0.14 ± 0.08 |
